# The Time has Come: Building an Effective Preventive Alliance Beyond Community Health Crisis

**DOI:** 10.3389/ijph.2021.637821

**Published:** 2021-03-26

**Authors:** Alberto Borraccino, Patrizia Lemma

**Affiliations:** Department of Public Health and Paediatrics, University of Torino, Torino, Italy

**Keywords:** health management organizations, health policy, strategic planning, knowledge translation, decision-making

Emergency conditions can reveal existing hidden organizational deficits or relationship difficulties. Di Ruggiero (2018) [1] invited scholars to discuss on the elements that influence the public health decision making process. Thereby, our aim is to propose a reflection on the potential mechanisms that favor or hamper knowledge translation for improving evidence-informed decision-making in public health, reflecting on the critical points that can hinder effective collaboration.

## Health Policies, Actors and Processes

The inability to build effective relationships among administrative/political leaders, who are tasked with making final decisions; scientific researchers in public health, who are charged with producing evidence-based knowledge; and health professionals working in the field, who are in constant contact with the population, has been reported to be one of the main causes of predictable, often foreseen errors that can impact the health of a community [2–5] and complaints are reciprocal [6].

Public health researchers and health professionals complain that the political choices influencing community health are seldom based on scientific evidence [6]. They also often complain of widespread political myopia, with politicians seeming to focus on investments that have only short-term effects, especially during election seasons. This approach does not encourage the collaboration needed to develop strategies with long-term goals [7]. On the other hand, health professionals and public health researchers rarely endeavor to communicate with politicians, which makes them poorly placed to find out when health-related political decisions are being made and reduces their ability to provide relevant information [4]. Therefore, politicians tend to avoid debates with public health researchers, out of a fear that, due to the inherent distance between their fields, the discussion would not give rise to useful practical proposals [6, 8].

The aforementioned recriminations demonstrate that politicians, public health researchers, and health professionals each occupy, in the ecological sense of the term, a “niche” that operates within its own paradigm, using its own idioms and internal codes of practice. These professional niches have developed despite the fact that the work cycles of these fields follow the same logical steps: problem identification, problem analysis, selection of suitable approaches to face the problem, program implementation, and evaluation of program impact. However, these work cycles do not necessarily evolve linearly, concluding each step before moving forward and avoiding backwards shifts [4, 6]. Those who conduct analyses repeatedly move back and forth helicoidally among the cycle steps in search of a higher level of agreement. Unfortunately, throughout this iterative evolution, each cycle step is embedded in the respective niche’s paradigm, and the differing idioms and practices complicate understanding among politicians, researchers, and health professionals, thus stifling collaborations. Moreover, difficulties arise because the criteria that need to be fulfilled to proceed to the next step of the work cycle are often acknowledged and implicitly already accepted within each of the three “niches” by cultural affinity, producing disconnections among them [6].

## The Working Cycles

In the political work cycle, the first step, problem identification, centers on the process through which a problem or theme becomes part of the political agenda. This first step is absolutely crucial and highly unpredictable; it is the result of a process of adjustment between how an idea is designed by the political elite, usually done through an ideological lens, and how the same idea is perceived by the general public. In both cases, politicians must deal with the role played by the media and social media in “shaping” the problem and presenting hypothetical solutions [9]. In this situation, researchers and health professionals cannot easily influence what is in the political agenda. Unfortunately, the three fields have their own, separate agendas. Once the issue becomes part of the political agenda, suitable solutions are generally the result of a lobbying process among stakeholders engaged in the political cycle. Therefore, the final political decision is necessarily the result of negotiation [6].

In the work cycle of health professionals, problems are shaped and agendas set to fulfill users’ needs. These problems are often identified by listening to requests that are directly brought by citizens and framed through health professionals’ knowledge and perceptions of what is desirable and achievable. Feasibility and immediacy tend to prevail both in the theoretical study of the problem and in the search for evidence of efficacy. Health professionals tend to embrace familiar solutions that come from their own experiences and studies, or solutions that have been previously employed. However, these solutions are often replicated without determining whether they will be effective in a context that is different from the original one. Moreover, solutions and programs are commonly developed without discussion with local political authorities and little attention to political legitimization, which can impact the feasibility of these programs. Political authorities or local administrators are only contacted once decisions have already been made by health professionals, without sharing the process that led to the decisions, and usually only when economic or organizational difficulties arise in implementing the decided actions. Impact evaluation is often decided at the end of the implementation process and mainly centers on satisfaction surveys administered to a few representatives of the affected community. Therefore, the real impact of these programs on the community health remains unmonitored, and this reducing both health professionals’ and politicians’ ability to defend their choices.

To collect evidence, public health researchers favor the use of specific experimental design approaches and meta-analytical syntheses to disseminate intervention guidelines. On the contrary, health professionals know first-hand that social change is the result of a complex interaction among environmental, organizational, cultural, and other factors that cannot be completely reduced to an experimental design. They also know that simply distributing scientific guidelines does not have the power to "transform the society", as such guidelines are rarely welcomed or immediately applied in the political and community arena [8].

## The Upstream Engagement: The Role of the Civil Society

According to Coveney (2010) [5], health professionals should better develop their “political acumen” to increase their likelihood of having a positive impact on community health as they not only are the link between the other social actors as we focus on here, but also because there are the main and the strongest link to the civil society, real drivers of the so called upstream engagement. Besides, recent studies have shown that non-expert judgment about risks has progressively gained value similarly as those of the experts. Indeed, shifting the decision-making process from the expert to the civil society assumes an important ethical value and provides more legitimacy of decisions [10, 11]. Only an improved relationship across health professionals and the civil society can help to pave the way for better monitoring of the impact of health policies, with a focus on accountability: namely on the process of explaining and justifying choices that are made [6].
